# Psychometric properties of the Malay version of the self-efficacy for exercise scale

**DOI:** 10.1371/journal.pone.0215698

**Published:** 2019-05-03

**Authors:** Abdulwali Sabo, Yee Cheng Kueh, Garry Kuan

**Affiliations:** 1 Unit of Biostatistics and Research Methodology, School of Medical Sciences, Universiti Sains Malaysia, Kelantan, Malaysia; 2 Exercise and Sports Science, School of Health Sciences, Universiti Sains Malaysia, Kelantan, Malaysia; São Paulo State University (UNESP), BRAZIL

## Abstract

**Background:**

The present study was aimed at validating the Malay-language version of the Self-Efficacy for Exercise Scale (SEE-M) using confirmatory factor analysis (CFA).

**Methods:**

Data were collected from undergraduate students at all campuses of the Universiti Sains Malaysia. A total of 1,605 students completed the SEE-M (female: 71.5%, male: 28.5%), with the mean age of 20.3 years (SD = 1.5). Perceived self-efficacy was assessed with the 18-item SEE-M. Standard forward–backward translation was performed to translate the English version of the Efficacy for Exercise Scale (SEE) into the Malay version (SEE-M).

**Results:**

The 2 initial measurement models tested (1-factor and 3-factor models) did not result in a good fit to the data. Subsequent investigation of the CFA results recommended some modifications, including adding correlations between the item residuals within the same latent variable. These modifications resulted in good fit indices for the 1-factor model (RMSEA = .059, CFI = .939, TLI = .922, SRMR = .049) and the 3-factor model (RMSEA = .066, CFI = .924, TLI = .903, SRMR = .051). The final measurement models comprised all 18 SEE-M items, which had significant factor loadings of more than .40. The test-retest results indicated that the SEE-M was stable, with an intra-class correlation of .99. The composite reliability was .886 for the 1-factor model and .670–.854 for the 3-factor model.

**Conclusions:**

The translated version of the SEE-M was valid and reliable for assessing the level of self-efficacy for exercise among university students in Malaysia.

**Perspective:**

This study examining the psychometric properties of the SEE scale based on CFA was the first to assess 2 proposed models (1-factor and 3-factor models) simultaneously and to translate the original, English-language SEE into Malay.

## Introduction

The primary, dynamic approaches that can be adopted to lower the risk of various chronic diseases (e.g., cardiovascular diseases, non-insulin-dependent diabetes mellitus, osteoporosis, obesity, and some cancers) can be achieved through regular participation in physical activity [1]. Regular exercise is an essential component of an effective, health-promoting lifestyle. For example, a 15-year prospective study verified that recreational physical activity is an independent predictor of a reduced cardiovascular mortality rate among adults. After adjusting for other risk factors (Framingham risk score and central obesity), the high recreational physical activity group was 35% less likely than the low recreational physical activity group to have cardiovascular mortality outcomes [2].

Although many researchers have recognized the benefits of exercise, an estimated nearly 3 million deaths and 32 million disability-adjusted life years annually are associated with physical inactivity globally [3]. Moreover, about 36% of Malaysian adults do not engage in physically active lifestyles. According to the Malaysian Adults Nutrition Survey, only 11%–15% of adults practice physically active lifestyles [4].

Pender [5] proposed the health promotion model (HPM), which integrates the psychological determinants of health behavior and can be used as a guide to develop exercise programs at the community and individual levels. The HPM describes the multidimensional characteristics of individuals’ socialization with their environment as they seek better health. The HPM integrates the self-efficacy construct from social cognitive theory [5].

Self-efficacy is persons’ belief in their capability to plan and carry out the course of action needed to achieve the given goals and to overcome any temptation to relapse. Self-efficacy is essential because individuals with high self-efficacy in a task strive harder to complete it and experience more positive emotions related to the task [6]. Self-efficacy is a key construct in social cognitive theory and has been utilised to describe the factors influencing exercise behavior [6].

In social cognitive theory, self-efficacy serves as a psychological instrumental construct associated with behavioral change [6]. Self-efficacy has been found to be a major predictor of the types of activities in which individuals choose to participate, the amount of effort spent during activities, and the persistence of behaviors and beliefs when confronting obstacles [7]. Successful people manage to sustain their dedication to accomplish challenging goals even when they encounter obstacles. They never worry about failure but see obstacles as tests to master, not threats to circumvent [8]. Zelle, Corpeleijn [9] confirmed that physical self-efficacy is a strong predictor of participation in and maintenance of physical activity and a significant mediator between fear of movement and physical activity among renal transplant recipients.

In contrast, persons who question their capability to succeed in challenging tasks may see tasks as threats and decide to avoid them due to personal weaknesses. Consequently, challenges might prevent them from succeeding [10]. Persons with low self-efficacy easily give up when confronted with obstacles and defeats and can quickly lose faith in their abilities. Studies have also proposed that various medical conditions, such as depression, can negatively influence exercise self-efficacy [11–13]. Factors linked to physical inactivity include medical problems, sedentary lifestyles, negative past experiences and related fears of activity, poor perceptions of physical activity, lack of socialization, and living in unsafe neighborhoods [14,15].

Many researchers [16,17], have attempted to assess persons’ levels of confidence in sustaining physical activity when confronted with challenges. The Self-Efficacy for Exercise Scale (SEE), the most commonly used scale, was first developed and verified as a valid, reliable instrument among older populations with a mean age of 85 years and SD of 6.2 [18], and has been administered to African American and Latino older adults in the United States [19]. The SEE has been translated into other languages, including Chinese [20], Korean [21] and Swedish [22], and the reliability and validity of these translated versions have been verified with samples of Taiwanese [23] and Swedish [24] populations. However, there is no version of the SEE in the Malay language.

There is a need to develop an effective measurement scale to assess levels of self-efficacy for participation in physical activity among university students, who are generally young adults. Previous studies [16,17], have demonstrated that improving self-efficacy plays a vital role in increasing the duration of physical activity among young adults. It, therefore, is imperative to identify the determinants that motivate and maintain physical activity participation. In this study, we aimed to translate the SEE into Malay for use with Malay populations and to confirm the reliability and validity of the SEE Malaysian (SEE-M) version among Malaysian university students.

## Method

### Participants

A total of 1,605 undergraduate students at the Universiti Sains Malaysia was recruited through convenience sampling. Larger samples commonly yield more robust results with greater replicability [25]. For this study, therefore, we considered a sample size of 1,605 to be adequate for confirmatory factor analysis (CFA) of the 18-item SEE-M. The participants included 457 male (28.5%) and 1,148 female (71.5%) students and had a mean age of 20.3 years (SD = 1.5). They identified themselves as Malay (80.1%), Chinese (11.7%), Indian (5.0%), and others (3.2%) but were all Malaysians and had sound reading and speaking skills in Malay. The median sessions of physical activity were 2 days per week, and the median duration of each session was 60 minutes. The most common sport activities were jogging, badminton, tennis, cycling, football, netball, and basketball.

### Questionnaire translation

The original English version of the SEE-M was translated into Malay through the following steps. First, the English version was translated into Malay by the third author, who aimed to maintain the contents’ meaning rather than render a literal, word-to-word translation. Second, the translated Malay version was back-translated into English by a local bilingual Malay. Third, these 2 versions were reviewed and finalized by a panel of 5 experts in sport sciences, health psychology, sport psychology, and physical education. The panel members were bilingual speakers in Malay and English and had more than 10 years of work experience in their fields of expertise. The panel reviewed the versions, relating each item to its corresponding item in the original English version. All the differences were properly corrected.

The contents were evaluated to determine if they were culturally suitable for Malaysian populations. Ten undergraduate students were invited to assess the clarity of the final Malay version. They were urged to respond to the items and give their views on the questionnaire’s contents and presentation. Their remarks were favorable and required no modifications. The translated Malay version is presented in the S1 Appendix.

### Data collection

The study was approved by the Universiti Sains Malaysia Human Research Ethics Committee and conducted in accordance with the Declaration of Helsinki. Data were collected from September to December 2018 on all campuses of the Universiti Sains Malaysia. The self-reported SEE-M questionnaire was used in this cross-sectional study. Before classes, the researchers approached lecturers and briefed them on the data collection method, and at the end of the classes, the students willing to participate remained in the classroom. They were given informational sheets on participation to read before being asked to complete the questionnaire. Implied consent was obtained when the participants volunteered to complete the questionnaire and returned it to the researchers. The estimated time to complete the SEE-M was 10–18 minutes.

The researchers collected 1,624 completed SEE-M questionnaires, including 1,605 with responses to all the items. Thus, the final sample was 1,605 questionnaires with no missing values. To measure the test-retest reliability of the SEE-M, 125 invited participants again completed and returned the questionnaire at day 14.

### Measures

#### Demographic, physical activity, and sports activities information

The questionnaire included items on the participants’ demographic characteristics (e.g., age, gender, and ethnicity), physical activity levels, sports participation, and hours per week spent engaged in physical activity or sports.

#### Self-efficacy for exercise scale

The SEE, as developed by Bandura [6], had only 1 factor, which accounted for 77.5% of the total variance [21]. Bandura [6] suggested that perceived self-efficacy does not directly measure the purpose of interest (i.e., evaluating general terms and not situational needs and circumstances.) but, instead, the object of interest (i.e., proficient performance); therefore, the scales related to perceived self-efficacy should be directed toward measures related to attaining the domain of functioning. Self-efficacy concerns perceived capability, so the items should be expressed as “can do” rather than “will do” and be composed at the participants’ level of understanding.

Shin, Jang, Pender [21], Kim [26], and Kosma [27] used a revised Korean version of the SEE with 3 factors. The revised scale consisted of 18 items, with a 5-point response scale from 1 (not at all confident) to 5 (extremely confident). The participants rated their level of confidence in their ability to perform exercise activities (i.e., at least 3 times per week) in different conditions, such as “when I am feeling depressed, or it is raining [27].” A 2-week, test-retest of reliability resulted in a reliability coefficient of .86 [26], and the 3 factors explained 96.4% of the total variance [21]. In this study, the Malay version of the SEE was examined with 1- and 3-factor models.

## Statistical analysis

Mplus version 8 was used to analyze the CFA results. The data were pre-screened, and those questionnaires with missing values were excluded from the analysis. The final data analysis included 1,605 completed questionnaires. The MLR Estimator was selected to perform CFA because it is robust to non-normality distribution of data and produces estimates with standard errors, including a mean adjusted chi-square statistic [28].

Two initial hypothesized measurement models with 18 observed variables (SEE-M items) were adopted and tested using CFA. In the first model, all the items were considered to be under 1 latent variable (1-factor model), while in the second model, the items were considered to be under 3 latent variables (3-factor model). Factor loadings of .40 and higher were considered to be significant and were used as criteria to retain or delete items from the measurement model [29]. Based on Hair, Black [30] recommendations for handling more than 12 observed variables (items) and sample sizes of more 250, the following fit indices and cut-point values were used: comparative fit index (CFI) and Tucker and Lewis index (TLI), with a recommended value of more than .92; root mean square error of approximation (RMSEA), with a recommended value of less than .07; and standardized root mean square residual (SRMR), with a recommended value of less than .08 [30]. The CFA modification index was also referred during model re-specification to obtain the best fit measurement models. The model were re-specified after the authors obtained adequate theoretical support.

After the researchers identified the best-fit measurement models for the 1- and 3-factor models, the construct validity of both was assessed. In CFA, construct validity has 2 components: discriminant validity and convergent validity. According to Hair, Black [30], convergent validity is a measure of the extent of the variance shared by the items in the same factor. Composite reliability (CR) and average variance extracted (AVE) were used to assess the scale’s convergent validity. CR was computed following Raykov’s method [31] in Mplus 8. The recommended values were greater than or equal to .60 for CR [32] and .50 for AVE [33]. Discriminant validity, or the degree to which a factor differs from other factors [34], was assessed by examining the correlations between the factors in the 3-factor model. A correlation coefficient of .85 or less between factors was regarded as acceptable discriminant validity [35].

To compare the present study with previous studies [21,26] on the SEE’s Cronbach alpha, we reported internal consistency reliability based on Cronbach’s alpha for the 1- and 3-factor SEE-M models. The SEE-M’s stability was also tested based on the subsample of 125 participants’ scores. Test-retest reliability based on intra-class correlation (ICC) was reported for the 1- and 3-factor models. Cronbach’s alpha and ICC were computed using SPSS 24.

## Results

### 1-Factor SEE-M measurement model

**Fig 1:**
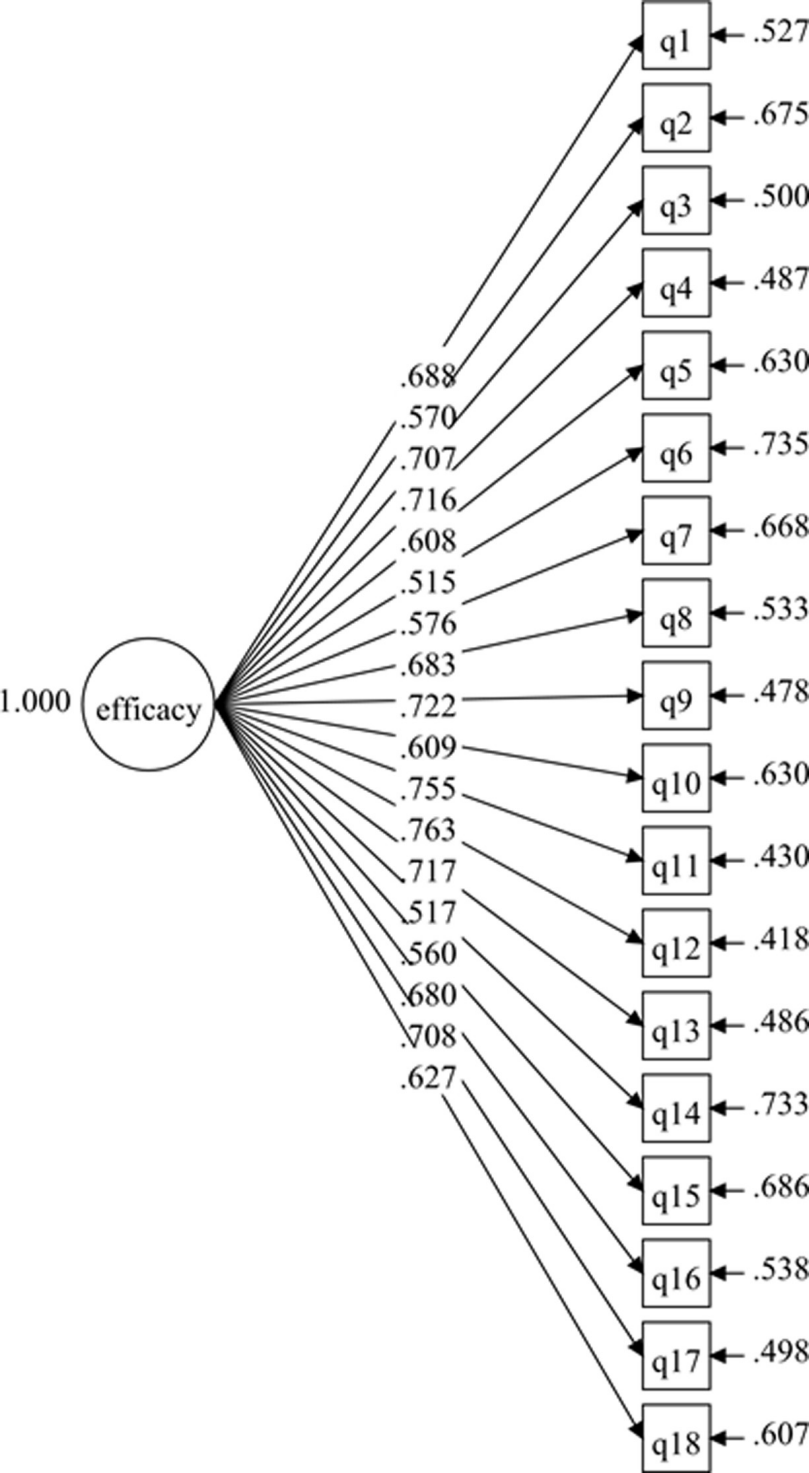
SEE-M measurement model (1-factor model 1).

**Fig 2:**
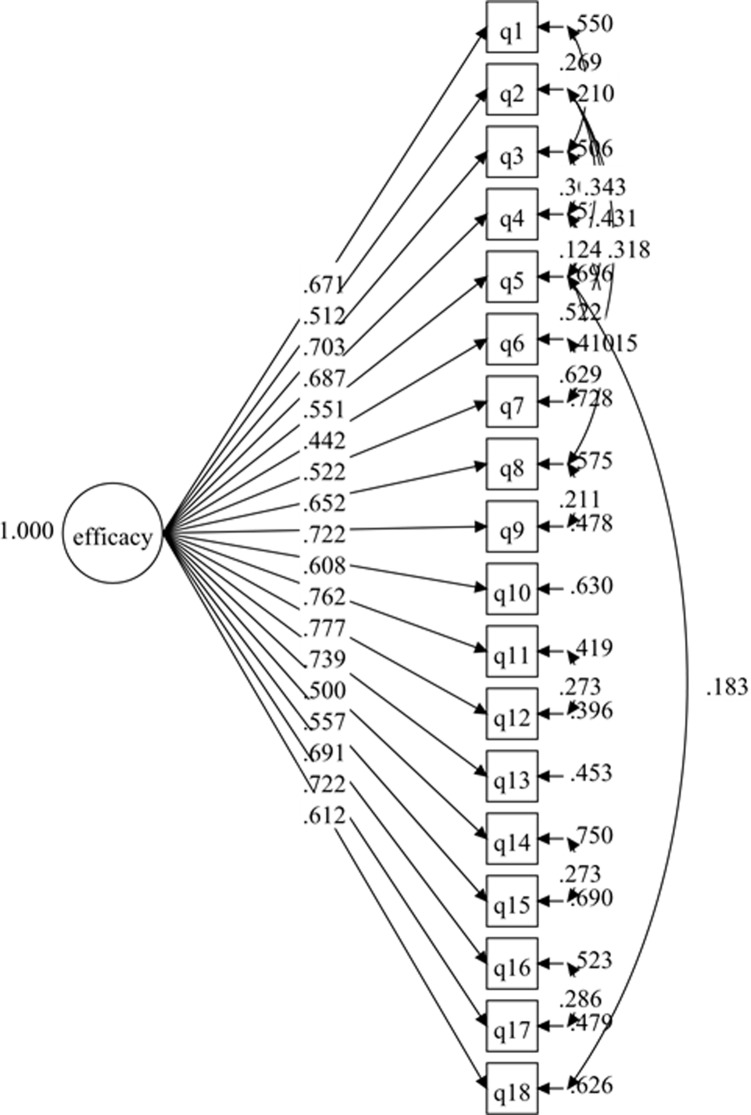
SEE-M measurement model (1-factor model 2).

The hypothesized measurement model for the 1-factor SEE-M consisted of 18 items within the same domain. The results for the initial hypothesized measurement model displayed poor fit to the data (Table 1), although the factor loadings of all the items were higher than .50 with p values < .001 (Fig 1). Further investigation improved the initial model by correlating the items’ residuals (Fig 2). The results of the second model showed good fit to the data (Table 1). The final model (1-factor model 2) was established without deleting any items after adding the correlations on the items’ residuals. In the results for the 1-factor model 2, the standardized item-loading model ranged from .442 to .777, which was regarded as acceptable to very good (Fig 2).

**Table 1:** Summary for 1-factor model fit indices.

Path model	RMSEA (90% CI)	CFI	TLI	SRMR
1-Factor Model 1	0.108 (0.105, 0.112)	0.768	0.737	0.079
1-Factor Model 2^a^	0.059 (0.055, 0.063)	0.939	0.922	0.049

^a^1-Factor measurement model with correlated items residual; Q7 andQ6, Q6 and Q5, Q7 and Q5, Q8 and Q4, Q4 and Q3, Q17 and Q16, Q12 and Q11, Q15 and Q14, Q2 and Q1, Q3 and Q1, Q6 and Q2, Q5 and Q2, Q7 and Q2, Q9 and Q8, Q18 and Q5, Q5 andQ4.

### 3-Factor SEE-M measurement model

**Fig 3:**
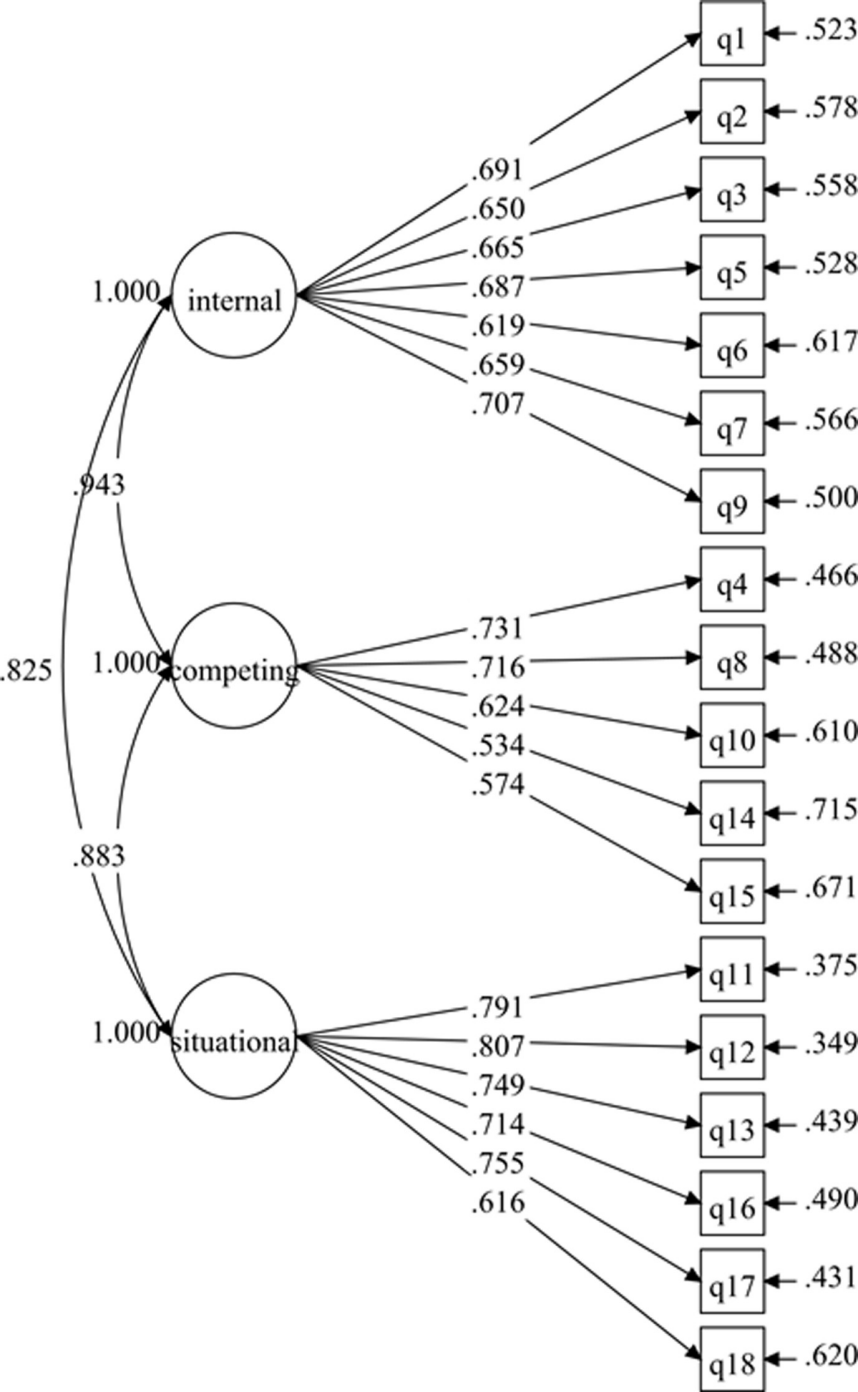
SEE-M measurement model (3-factor model 1).

**Fig 4:**
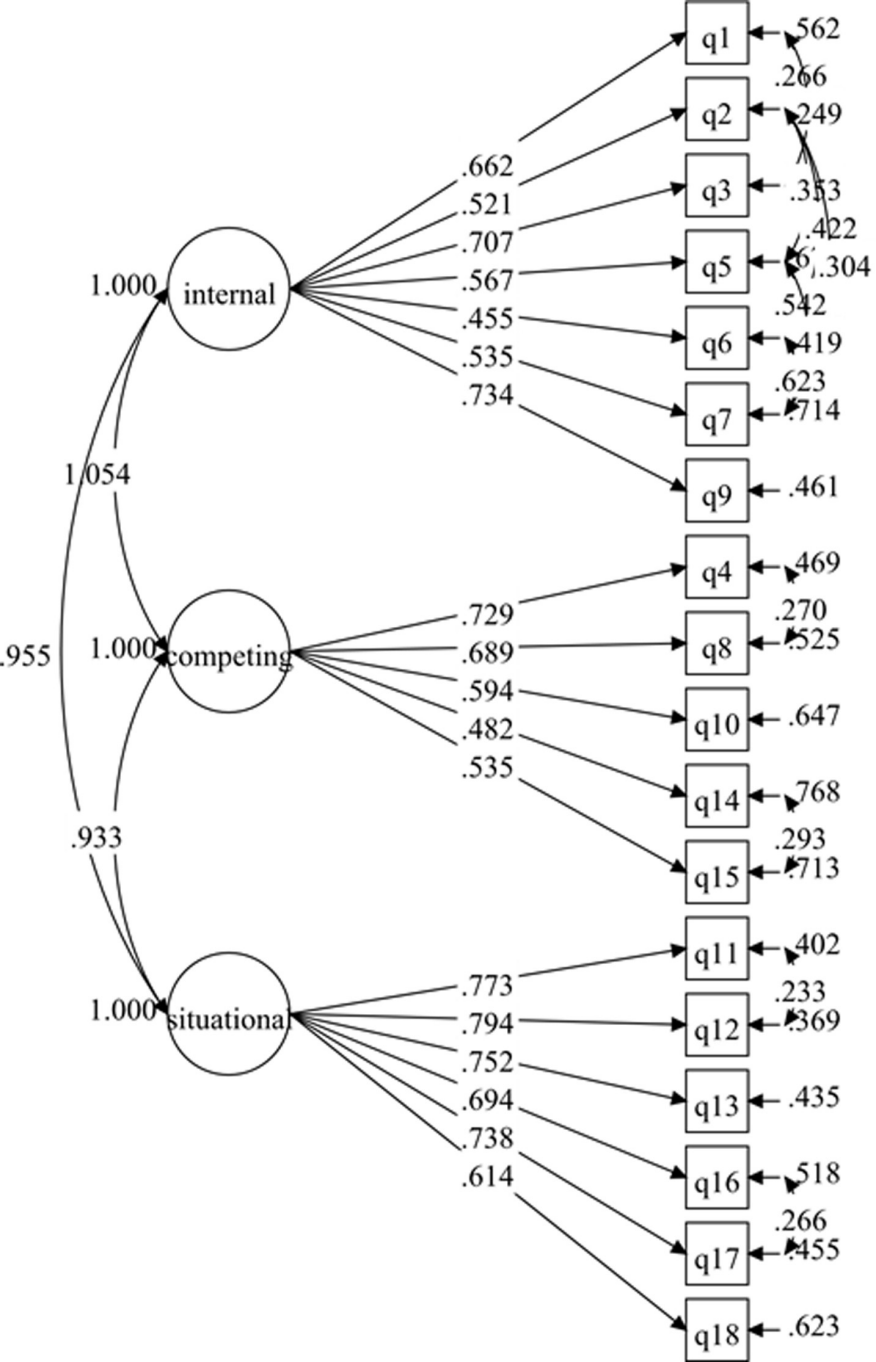
SEE-M measurement model (3-factor model 2).

The hypothesized measurement model for the 3-factor SEE-M consisted of 18 items and 3 factors: (1) situational/interpersonal (6 items); (2) competing demands (5 items); and (3) internal feelings (7 items) [21]. Testing of the initial hypotheses measurement model displayed poor fit to the data (Table 2), although the factor loadings of all the items were more than .50 with p-values < .001 (Fig 3). Further investigation improved the initial model by correlating the items’ residuals within the same latent variable (Fig 4). The resulting re-specified model fit the data well (Table 2). The final model (3-factor model 2) was confirmed without deleting any items after adding the covariances for the correlated items’ residuals within the same latent variable. The results from 3-factor model 2 revealed that the standardized item-loading model ranged from .455 to .794 (Fig 4), which was regarded as acceptable to very good.

**Table 2:** Summary for 3-factor model fit indices.

Path model	RMSEA (90% CI)	CFI	TLI	SRMR
3-Factor Model 1	0.103 (0.100, 0.107)	0.795	0.762	0.076
3-Factor Model 2^a^	0.066 (0.062, 0.070)	0.924	0.903	0.051

^a^3-Factor measurement model with correlated items residual of the same domain; Q7 and Q6, Q6 and Q5, Q7 and Q5, Q15 and Q14, Q17 and Q16, Q6 and Q2, Q8 and Q4, Q5 and Q2, Q7 and Q2, Q2 and Q1, Q3 and Q1, Q12 and Q11

When comparing the final 1- and 3-factor models, 1-factor model 2 had greater CFI and TLI values and lower RMSEA and SRMR values than 3-factor model 2. Based on the present data, these results indicated that the 1-factor model was more desirable than 3-factor model.

### Convergent and discriminant validity

The CR was .886 for the final 1-factor model and .670–.854 for the final 3-factor model. The AVE was .430 for the final 1-factor and .268–.457 for the final 3-factor model. Although the AVE values were less than the prescribed value of .50, all the CR values were more than the recommended value of .60. The 2 models, therefore, were considered to have adequate convergent validity [33]. Table 3 presents the CR and AVE values and the correlation coefficients for the final 3-factor model.

**Table 3:** Composite reliability (CR), average variance extraction (AVE), and factor correlation for 3-factors final model for SEE-M.

Variables	CR	AVE	1	2	3
1. Internal feeling	.670	.366	1	.638^*^	.622^*^
2. Competing	.780	.268		1	.689^*^
3. Situational	.854	.457			1

*Correlation is significant at the 0.05 level (two tailed).

### Test-retest reliability

For test-retest reliability, 125 participants volunteered to complete the SEE-M again at day 14 after completing it at the first session. Their mean score was 48.40 (SD = 13.0) at day 1 and 48.40 (SD = 13.3) at day 14. The ICC based on the 1-factor model was .990 (95% CI, .986, .993, p value < .001), while based on the 3-factor model, the ICC was .848 for internal factor (95% CI, .790, .891, p value < .001), .751 for competing factor (95% CI, .664, .819, p value < .001), and .838 for situational factor (95%CI, .776, .883, p value < .001). These results indicated an excellent, stable ICC [36].

### Internal consistency

For the 1-factor SEE-M model, a Cronbach’s alpha of .931 was obtained indicating that the measurement was highly reliable. For the 3-factor SEE-M model, the Cronbach’s alpha was .852 for internal factor, .774 for competing factor, and .877 for situational factor.

## Discussion

In this study, we translated the English version of the SEE into Malay and then confirmed the questionnaire’s psychometric properties to provide a tool to measure individuals’ levels of self-efficacy for participation in physical activity among Malay-speaking populations. Perceived self-efficacy is an essential behavior in the causal mechanisms that influence the health promotion achieved through lifestyle modifications [5]. Consequently, the SEE items should accurately reflect the self-efficacy construct and be tested with all ethnic and racial populations.

The outcomes of perceived self-efficacy can be examined through its various influences on motivation, belief, interest, and behavior. Construct validation enables continuously evaluating the validity of the hypothesized causal structure in the conceptual design and self-efficacy scales [6]. Bandura [6] original self-efficacy scale with a 1-factor model was tested among Korean adults with chronic diseases. The results showed that the 1-factor model explained 77.5% of the total variance, and the 3-factor model explained 96.4% of the total variance [21].

The SEE-M with 1- and 3-factor models tested in this study proved to have internal stability with the sample studied. The Cronbach’s alpha of .931 for the 1-factor model obtained matched those found in other study, such as .94 reported by Shin, Jang [21], and .91 reported by Kim [26]. The corrected item–total correlation for all items was higher than .46. According to Nunnally and Bernstein [37], item–total correlations of less than .3 are considered to be insufficient and indicate that items do not contribute to the measurement of the main factor. The corrected item–total correlation demonstrates that the SEE-M scale has good internal consistency. The test-retest reliability was .990, and a mean of 44.80 was obtained at both the first and the second measurements, with standard deviations of 13.0 and 13.3, respectively. These results indicate that the SEE-M scale remains highly stable over time. Previous studies on the SEE scale have reported 2-week test-retest reliability coefficients of .86 [26] and .86 [21].

This study’s CFA results confirm that the 1- and 3-factor SEE-M models had good fit with the data obtained from Malaysian undergraduate students as no items were deleted from the original version. These findings show that the self-efficacy construct in the both the 1- and 3-factor models of the SEE-M was consistent with earlier studies based on the sample studied [6,21,26]. However, the 1-factor model appeared to perform relatively better than the 3-factor models based on several fit indices. The correlation values of the 3-factor model were all less than the prescribed value of .85, suggesting that it has good discriminant ability [35].

CFA was performed to investigate and establish the factor model of SEE-M by evaluating the measurement model validity based on the initial measurement theory. The SEE-M was hypothesized to contain 18 items in a 1-factor model and was later tested with a 3-factor model comprised of the same items grouped in different subscales. After establishing the 1- and 3-factor models of SEE-M, we further evaluated their construct validity, or the degree to which the items revealed the proposed theoretical factors within a construct; in other words, we estimated the precision of the measurement [30]. The construct validity of the SEE-M was assessed based on convergent validity and discriminant validity. The estimated AVE, or the average of the squared factor loadings for each subscale, was .268–.457. When AVE values are less than .50, the construct still might have adequate convergent validity if the CR is more than .60 [33,38]. The construct reliability obtained in this study using CR was .670–.886, greater than the recommended level of .60 [32]. In addition, the correlations between the factors in the 3-factor model were all less than the prescribed value of .85. These results suggest that the subscales in 3-factor SEE-M model are unique, the factors do not overlap much, and each factor explains different variance than the other factors. The results confirm previous findings that the 3-factor model explains an additional 18.9% of the total variance than the 1-factor model [21].

Perceived self-efficacy for physical activity has been found to be an important predictor of participation in physical activity [39]. It has also been suggested that middle-aged adults who possess high levels of self-efficacy are more inclined to engage in physical activity in spite of many barriers and may be more enthusiastic about physical activity than their peers with low self-efficacy [40]. It, therefore, is helpful to use the SEE-M to assess individuals’ levels of perceived self-efficacy for participation in physical activity. The information obtained can serve as a guide to develop interventions that promote physical activity participation through boosting self-efficacy. The SEE-M can be used by professionals in Malaysia, such as physical educators, psychologists, and other health care providers, to understand the relationship of self-efficacy to participation in physical activity among their Malay-speaking clients.

Among the limitations of this study, data were obtained from a single university, which might have limited the generalizability of the results to other university students. However, the large sample size strengthened the conclusions and findings. Another limitation was the use of a self-reported, paper-based survey. The self-reported measures could have given rise to response bias, which may have reduced the accuracy of the obtained data. In addition, the respondents could have been subjected to social desirability and consequently answered the items in ways to reflect well on themselves [41]. The participants, though, were aware that the researchers could not identify them, and their names were not included in the questionnaire, which could reduce the possibility of such responses. The participants were also assured of the confidentiality of the data and urged to answer honestly to all the items related to their self-efficacy for participating in physical activity.

The present study confirmed that the SEE-M with 1- and 3-factor models with no items deleted has good construct validity, consistent with previous studies on the SEE. Nevertheless, it is essential to further examine the replicability of SEE-M in different populations with diverse ages, education levels, occupations, and health conditions. The 1- and 3-factor models of the SEE-M could also be fruitfully studied in more diverse Malay-speaking populations.

### Conclusion

The final measurement model for the SEE-M tested with 1- and 3-factor models has been shown to be a valuable measurement tool for evaluating exercise beliefs. All the items were retained and confirmed to be fit for the sample data. Based on the reported fit indices, the 1-factor model outperformed the 3-factor model. However, given that both models fit the data well, we recommend both as acceptable for use in measuring individual’s self-efficacy for exercise. The choice depends on whether the researchers want to interpret the SEE score as a single score or as separate scores for 3 domains (internal feelings, competing demands, and situational/interpersonal). Researchers, sport psychologists, and exercise educators can use the SEE-M to measure levels of self-efficacy for exercise among people whose main spoken language is Malay.

## Supporting information

S1 Appendix The malay version of the self-efficacy for exercise scale. (DOCX)

S1 Data SEE-M data for CFA. (DAT)

S2 Data SEE-M data for test-retest. (DAT)
