# Supplementary material for: Psychometric properties of the Malay version of the self-efficacy for exercise scale
Source: PLoS One. 2019 May 3;14(5):e0215698. doi: 10.1371/journal.pone.0215698 (PMC6499426; doi:10.1371/journal.pone.0215698)
Supplement: S1 Appendix — (DOCX) [file pone.0215698.s001.docx]

**The Malay Version of the Self-Efficacy for Exercise Scale**

**Skala Keberkesanan Diri Untuk Senaman**

Beberapa situasi yang diterangkan di bawah adalah situasi yang menyebabkan kesukaran untuk mengekalkan senaman secara rutin. Sila tanda dalam setiap kotak di bawah seberapa pasti anda yakin dapat melakukan rutin senaman secara kerap (tiga atau lebih kali seminggu). Tandakan **( / )** mengikut tahap keyakinan anda.

|  |  | Tidak yakin  sepenuhnya  (1) | Agak yakin  (2) | Sederhana  yakin  (3) | Sangat yakin  (4) | Benar-benar yakin  (5) |
| --- | --- | --- | --- | --- | --- | --- |
| 1. | Apabila saya berasa letih |  |  |  |  |  |
| 2. | Apabila saya berasa tertekan dengan kerja |  |  |  |  |  |
| 3. | Semasa cuaca buruk |  |  |  |  |  |
| 4. | Selepas pulih daripada kecederaan yang menyebabkan saya berhenti bersenam |  |  |  |  |  |
| 5. | Semasa atau selepas mengalami masalah peribadi |  |  |  |  |  |
| 6. | Apabila saya berasa tertekan |  |  |  |  |  |
| 7. | Apabila saya berasa bimbang |  |  |  |  |  |
| 8. | Selepas sembuh daripada sakit yang menyebabkan saya berhenti bersenam |  |  |  |  |  |
| 9. | Apabila saya berasa ketidakselesaan fizikal ketika bersenam |  |  |  |  |  |
| 10. | Selepas percutian |  |  |  |  |  |
| 11. | Apabila saya terlalu banyak kerja di rumah |  |  |  |  |  |
| 12. | Apabila ada pelawat |  |  |  |  |  |
| 13. | Apabila ada perkara lain yang menarik untuk dibuat |  |  |  |  |  |
| 14. | Jika saya tidak mencapai matlamat senaman saya |  |  |  |  |  |
| 15. | Tanpa sokongan daripada keluarga dan rakan-rakan |  |  |  |  |  |
| 16. | Semasa percutian |  |  |  |  |  |
| 17. | Apabila saya ada masa komitmen yang lain |  |  |  |  |  |
| 18. | Selepas mengalami masalah keluarga |  |  |  |  |  |
